# Negro Traits

**Published:** 1902-02

**Authors:** E. G. Ferguson

**Affiliations:** Macon, Ga.


### NEGRO TRAITS.*

BY E. G. FERGUSON, M.D.
MACON, GA.

Since President Roosevelt has entertained Booker Washington, the Alabama negro schoolteacher, at the White House the negro or race question has been revived throughout the country and in this connection the characteristics of the negro may prove of much interest to many people who have never made a study of them. The black skin, as a matter of course, marks them from all other nationalities. There is the receding forehead and chin, with black kinky hair, thick lips, flat nose, large open nostrils, so much so that you can look into them as you can a cow or horse, large eyes; in the male, beardless face; a straight-edge placed upon the face will not reach either the chin or nose at the same time before touching the lips. The arms are longer than in the Caucasian and bear a close resemblance to the order of ape, chimpanzee or gorilla. The foot has no arch, but where it ought to be arched is lower than the heel and toes so as to resemble a rocker, and in walking this imparts a motion peculiar to the race. While living in a city they will invariably seek earth to walk on in preference to pavements. The negro has protruding umbilicus or navel, closely resembling the cow, more so

*Read Before the Macon Medical Society.

than any other animal, while in the Caucasian it is retracted and drawn within itself so as not to be manifest except as an indentation on the abdomen. When a gang are at work there will always be one or more looking around as if in fear of approaching danger. If you are having your shoes shined the nigger will rub a little and look off from his work as though something might escape his observation or take him unawares. Like a flock of geese feeding in a pond there is always one on the watch to warn the others of danger. Like the horse, cow or dog, there is no cerumen or wax in his ear. He has small calves to his legs, the gastrocnemii muscles are without development. In a race with the Caucasian he is invariably overtaken, as the power of these muscles assert themselves in the Caucasian and are deficient in the Ethiopian. Who has ever heard or known of a negro man or woman fainting at any horrible sight as white men often and women invariably do? He has no sympathy for his fellow man or for the beast he uses. To him it has no feeling or requires no care. So long as a horse answers his purpose he employs the brute, and when it is of no longer a service from neglect, misuse and starvation he turns it out to seek its living as best it can. He is monkey-like and imitates all he sees. He rarely misses anything exposed to his view without repeating it as perfectly as his powers of imitation will permit. These are of a limited character, and often he makes the most laughable and egregious mistakes. Without the least compunction of conscience, he betrays the most sacred trust reposed in him. He has no regard for the truth, and when the truth would answer his purpose best he will lie. Is without gratitude or appreciation of anything done for him. Like the pet crow he is a natural born thief. If chance offers he will steal anything, no matter how worthless it may be to him. Virtue is unknown to him. He has no morals. Turpitude is his ideal of all that pertains to life. He can be educated to a certain degree, but not beyond. You cannot make a cocoanut hold any more milk than is in it, then why expect to elevate him to the standard of the Caucasian when there is no brain to cultivate. He is careless and negligent of his person and all his surroundings, quite willing to live in squalidness and want all of his life. His progeny are illy provided for at home and are allowed to roam at large, without restraint, and seek subsistence as best they can, growing up like any animal, as is well known to all familiar with this portion of the brute creation. Knocks and kicks are his persuasive eloquence in giving guidance to the offspring of his household. As yet no case of genuine sunstroke has ever been recorded against him. Heat seems to have little effect on his physical structure. His favorite place to sleep is in full exposure to the sun's rays. Is fond of alcoholic stimulants, but as a general thing rarely goes to excess. The teeth are even, white and of a pearly lustre, which may be owing to the contrast the black background affords. They rarely decay before advanced years. The quantity of food consumed is small in comparison to the white race and he is fonder of the coarser articles than any of the more civilized dishes. If left to himself will roam about all night and sleep all day. The exhalation from the skin is as characteristic as the kinky hair and black skin, even although frequent baths are indulged in, which is the exception in place of the rule, without in any manner reducing the effluvium. So great is this that after leaving a room where he has been for a few minutes the odor will remain for hours.

---

DR. R. S. HUIDEKOPER died at Philadelphia on the 17th of December, aged forty-eight. Dr. Huidekoper was a graduate of the University of Pennsylvania, and the founder of the Veterinary School. He was one of the most distinguished veterinarians in America. During the Spanish-American War he was surgeon-in-chief at Chickamauga, and although one of the ablest medical officers in the army medical service, he came in for an unusual amount of popluar abuse. His great distinction in veterinary medicine caused his name to be bandied about as a "horse doctor."

